# Comparative *in vitro* cytotoxicity and binding investigation of artemisinin and its biogenetic precursors with ctDNA†

**DOI:** 10.1039/d0ra02042g

**Published:** 2020-06-25

**Authors:** Neha Maurya, Khalid Imtiyaz, M. Moshahid Alam Rizvi, Khaled Mohamed Khedher, Prashant Singh, Rajan Patel

**Affiliations:** Biophysical Chemistry Laboratory, Centre for Interdisciplinary Research in Basic Sciences, Jamia Millia Islamia New Delhi-110025 India rpatel@jmi.ac.in +91 11 26983409 +91 8860634100; Department of Biosciences, Jamia Millia Islamia New Delhi-110025 India; Department of Civil Engineering, College of Engineering, King Khalid University Abha 6421 Saudi Arabia; Department of Civil Engineering, ISET, DGET Nabeul Tunisia; Department of Chemistry, ARSD College, University of Delhi New Delhi-110021 India

## Abstract

Artemisinin (ART) and its biogenetic precursors artemisinic acid (AA) and dihydroartemisinic acid (DHAA) are important traditional medicinal herb compounds with tumor growth inhibition properties. Herein, we have studied the cytotoxicity of ART, AA, and DHAA on different cancer cell lines (H1299, A431, and HCT 116) and investigated in detail their binding mechanisms with ctDNA by using spectroscopy, cyclic voltammetry, and computational methods. The UV absorbance, cyclic voltammetry, DNA helix melting, competition binding, and circular dichroism studies suggested that the complex formation of ART–ctDNA and AA–ctDNA occurs through groove binding. However, in the case of DHAA–ctDNA interaction, electrostatic interaction plays a major role. The thermodynamic parameters, *viz.*, Δ*G*^0^, Δ*H*^0^, and Δ*S*^0^ were calculated, which showed the involvement of hydrogen bonds and van der Waals interactions for drug–ctDNA interaction. FTIR and molecular docking results suggested that ART, AA, and DHAA were bound to the A–T rich region in the minor groove of ctDNA.

## Abstract 2

Artemisinin (ART) and its biogenetic precursors artemisinic acid (AA) and dihydroartemisinic acid (DHAA) are important traditional medicinal herb compounds with tumor growth inhibition properties.

## Graphical abstract

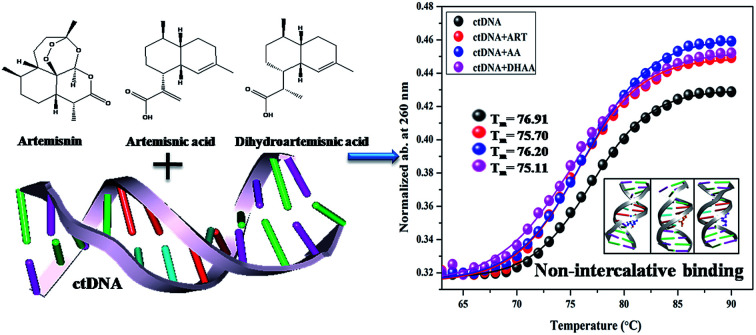

## Introduction

Nucleic acids such as DNA are the pharmacological target of anticancer drugs that are currently used in pharmaceutical development and advanced clinical trials. The binding interaction of anticancer drugs with DNA provides significant changes in the DNA properties and gene expression; this alteration influences the cell proliferation and has a great impact on the physiological functions of the cancer cell. Therefore, the binding mechanisms of drugs and DNA are essential for predicting different parameters required for chemotherapeutic drugs to control the disease. In the last few decades, drug–DNA interaction has been a hot research subject, which has a decisive role in understanding the molecular mechanism replication and transcription processes for the treatment of cancer, and are also used to design new anticancer drugs.^1–4^ It is evident from the literature that the drug freely interacts with the double helical structure of DNA through three major binding modes: (i) intercalative binding, wherein the drug is stacked in between the base pairs of the nucleic acid, (ii) groove binding, wherein the drug binds in the minor groove or major groove of the DNA helix with van der Waal's interaction, and (iii) electrostatic binding between positive charges of the drug and the negatively charged DNA phosphate backbone.^5,6^ Moreover, some alkylating and cross-linking agent also directly damage the DNA such as metal complexes.^7–9^

Artemisinin is a sesquiterpene lactone with a peroxide group extracted from the Chinese herb sweet wormwood (*Artemisia annua*). Artemisinin was isolated for the first time from the extracts of sweet wormwood.^10–12^ Generally, artemisinin is used for the treatment of malaria as a first line drug. However, in the last two decades, artemisinin and its derivatives have been recognized as anticancer agents and have enviable antitumor activities with low toxicities that can enable them to become chemo-preventive agents in cancer therapy. Artemisinin and its derivatives inhibit a range of cancer cells such as breast cancer,^13^ leukemia,^14^ melanoma,^15^ brain glioma,^16^ head and neck carcinoma,^17^ liver cancer,^17^ lung cancer, ovarian cancer, prostate carcinoma,^18^ B cell lymphoma, cervical cancer, pancreatic cancer,^19^ and nasopharyngeal cancer.^20^ Artemisinin and its derivatives are endoperoxide drugs that act either directly by inducing oxidative DNA damage or indirectly by interfering with the signaling pathways and apoptosis mechanism.^12^ Artemisinic acid and dihydroartemisinic acid are potential biogenetic precursors for artemisinin, which are extracted from the same plant species. Structurally, they are much simpler than artemisinin and have a high potency to inhibit tumor cells.^21^ Artemisinin and its biogenetic precursors can be administered in our body in many ways such as orally, intramuscularly, intravenously, and rectally. Due to the presence of the endoperoxide moiety, they act as free radicals by generating alkylating carbon-centered radicals, which results in DNA damage.^22^ Because of their great potential to inhibit cancer cells, researchers have explored their binding with biomolecules. The interaction of these types of natural products with DNA provides a new platform to develop new and effective drugs against cancer. Consequently, the quest for new and efficient anticancer drugs and their interaction with various biological targets are crucially significant as the experimental outcome will further aid in their clinical trial and the further modification of their structure as clinically needed. The binding mechanism of artemisinin and its derivatives with biomolecules is yet to be unraveled.^23,24^ Therefore, a detailed study of the interaction of artemisinin with ctDNA will be enormously significant.

In the present study, we have shown the cytotoxic effect of artemisinin and its biogenetic precursors and explored their binding mechanisms with ctDNA using spectroscopy and computational methods. Absorption spectroscopy, competitive binding, helix melting, circular dichroism, and FTIR were utilized to determine the binding strength, binding mode, and structural aspects of the drug (ART, AA, and DHAA)–ctDNA interaction. In addition, electrochemical measurements were also done to observe the variation in the electrical potential of drug–ctDNA binding. Molecular docking was also performed to show the binding position of the drugs in ctDNA. The finding of the present work might help to explore the binding efficiency of artemisinin and its biogenetic precursors to its major target, which is very crucial for the cancer research field.

## Results and discussion

### MTT assay

**Fig. 1:**
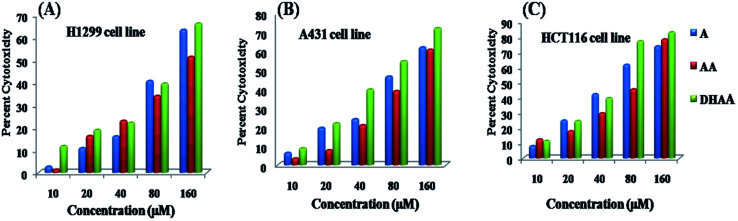
Cytotoxicity evaluation of ART, AA, and DHAA through the MTT assay after 48 hours of treatment on (A) HCT 116, (B) A431, and (C) H1299 cell lines.

The antiproliferative property of three bioactive compounds, namely, artemisinin (ART), artemisinic acid (AA), and dihydroartemisinic acid (DHAA) have been investigated and the potential anticancer cytotoxic activity against three cancer cell lines, namely, the lung cancer cell line H1299, the human skin cancer cell line A431, and the colon cancer cell line HCT 116 was compared through the MTT assay. The result signifies that ART, AA, and DHAA showed concentration dependent response (10–160 μM), as shown in Fig. 1. The cytotoxicity results were evaluated through cell growth inhibition, which are expressed as IC_50_ values (occurrence of 50% cell death at that concentration).^25,26^ IC_50_ was determined using the plots of cytotoxicity *versus* the concentration of the drugs and are tabulated in Table 1. Table 1 specifies that artemisinin and its biogenetic precursors show the most potent antiproliferative effect against the HCT116 cell line with the lowest IC_50_ value in comparison to that for the H1299 and A143 cell lines. Comparatively, the IC_50_ values of artemisinin and its biogenetic precursor DHAA was lower than that of ART and AA under identical experimental conditions. This result suggested that DHAA has more cytotoxic effect than ART and AA, and hence, it may be utilized and provide a great impact in the treatment of colon cancer.^14^

**Table 1:** IC_50_ values of artemisinin, artemisinic acid, and dihydroartemisinic acid on three cancer cell lines, *viz.*, A431, HCT 116, and H1299

Drugs	IC_50_		
	H1299	A431	HCT 116
ART	119.95 μM	113.74 μM	59.52 μM
AA	146.40 μM	127.81 μM	92.78 μM
DHAA	113.47 μM	67.41 μM	50.49 μM

### Cyclic voltammetry study

**Fig. 2:**
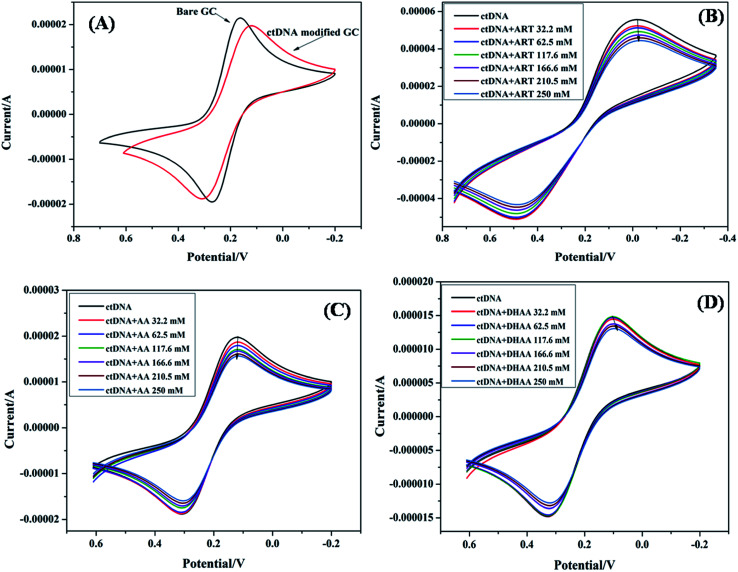
CV spectra of ctDNA (50 μM) with bare electrode (A) and in the presence of different concentrations of ART (B), AA (C), and DHAA (D).

Electrochemical methods such as cyclic voltammetry are imperative to evaluate the binding strength and mechanism of drug–DNA interaction under the physiological conditions.^27–29^ The change in the peak current and peak potential suggested that the complex formation between the drug and DNA, and is useful for determining the binding parameter and binding modes such as intercalative, groove binding, and electrostatic interactions. In general, the peak potential shifted to a more negative value corresponding to the electrostatic interaction while a more positive shift was observed owing to intercalative binding.^30^ Groove binding represents a less positive or no shift in the potential peak.^31^ Consequently, herein, we have utilized cyclic voltammetry to study the binding mode and binding parameter between the drugs (ART, AA, and DHAA) and ctDNA at a glassy carbon electrode. The ctDNA modified electrode and a K_3_Fe(CN)_6_/K_4_Fe(CN)_6_ electrolyte probe were used for characterization because of the non-electrochemical properties of the drugs and ctDNA. Fig. 2A shows the electrochemical response with a reversible redox peak of bare glassy carbon electrode and ctDNA modified electrode in K_3_Fe(CN)_6_/K_4_Fe(CN)_6_ solution. From Fig. 2A, it can be observed that the ctDNA modified electrode shows a great deviation in the redox peak current and peak potential. The peak potential of the electrode decreased from 0.16 V to 0.11 V and the peak current decreased from 2.18 × 10^−5^ to 1.97 × 10^−5^ A, respectively. This suggests that the ctDNA acted as an electron and mass transfer blocking layer on the electrode surface, which resulted in a decrease in the electron transfer rate of K_3_Fe(CN)_6_/K_4_Fe(CN)_6_ and hindered the diffusion rate of K_3_Fe(CN)_6_/K_4_Fe(CN)_6_ towards the glassy carbon electrode surface. This result indicates the modification of the glassy carbon electrode surface by ctDNA. The ctDNA modified electrode was used as a working electrode for studying the drug and ctDNA interaction, where K_3_Fe(CN)_6_/K_4_Fe(CN)_6_ solution was used as the electrolyte. As we added the drug solution into the electrolyte, the redox current peak of modified ctDNA decreased in the presence of ART (5.5 × 10^−5^ to 4.4 × 10^−5^ A), AA (1.9 × 10^−5^ to 1.5 × 10^−5^ A), and DHAA (1.5 × 10^−5^ to 1.3 × 10^−5^A) (Fig. 2B, C and D, respectively). This decrease in the redox current peak arose due to the interaction of ctDNA with the drug on the electrode surface, which increased the density of the ctDNA film and reduced the diffusion coefficient of K_3_Fe(CN)_6_/K_4_Fe(CN)_6_ ions; due to this, the migration of ions through the film was hindered. The redox peak potential shows a minute shift in the presence of ART (0.018 to 0.019 V) and AA (0.119 to 0.117 V); however, a slightly negative shift was observed for DHAA (0.103 V to 0.084 V). These results suggest that ART and AA bind with ctDNA through groove binding and DHAA interacts with ctDNA *via* electrostatic interactions.^30,32^ In addition, the decrease in the peak current with increasing concentrations of the drugs shows that ART, AA, and DHAA interact with ctDNA in a concentration-dependent manner.

**Fig. 3:**
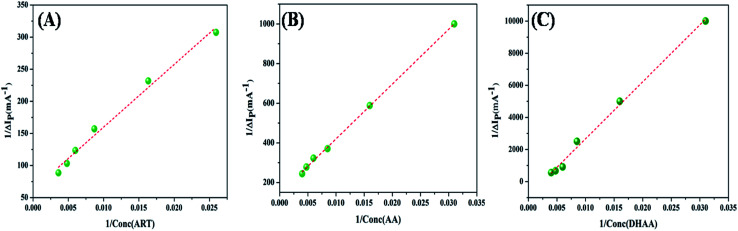
Linear relationship between the reciprocal of current drop *vs.* the reciprocal of ART (A), AA (B), and DHAA (C) concentration.

To calculate the binding parameter, we applied the Langmuir equation as follows and plotted the curve between the reciprocal of the current drop *vs.* the reciprocal of the drug concentration:^27^1
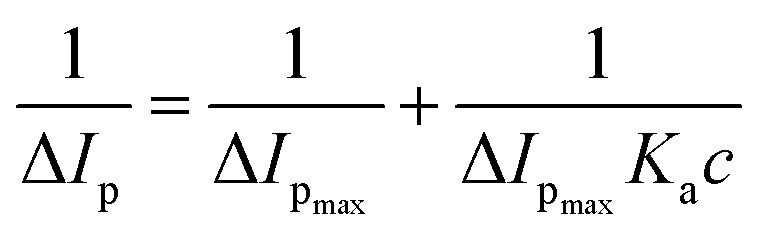
where, *K*_a_ is the binding constant of the drug–ctDNA interaction, and Δ*I*_p_ and Δ*I*_p_max__ are the current drop and maximum current drop (represented as concentration of drugs), respectively. Fig. 3 demonstrates the good linear relationship and slope of the curve (*K*_a_). The values of *K*_a_ for the drug–ctDNA interaction are summarized in Table 2. The overall results of the electrochemical studies suggest that ART and AA, and DHAA interact with ctDNA through groove binding and electrostatic interactions, respectively. This result was further confirmed by using the following spectroscopic result.

**Table 2:** Binding constants for ART, AA, and DHAA interaction with ctDNA using cyclic voltammetry

Drug	Binding constant (*K*_a_)
ART	9.76 × 10^3^
AA	2.76 × 10^3^
DHAA	3.55 × 10^2^

### Absorbance spectra measurement

Absorption spectroscopy is a well-known, effective, and one of the simplest techniques to observe the binding mechanism between a drug and DNA. Generally, the change in the absorbance such as the hyperchromism and hypochromism is observed when a small molecule interacts with DNA and results in structural alteration in the DNA. The hyperchromism that is observed due to the breakage of the duplex helical DNA structure generally corresponds to the electrostatic interaction,^33^ whereas hypochromism with a bathochromic shift shows the stabilization of the duplex structure by intercalative binding of the small molecule;^34^ however, groove binding is differentiated by an insignificant shift in the absorption spectrum.^35^ The interaction of ART, AA, and DHAA with ctDNA was examined by the electronic absorption spectrum of ctDNA (50 μM) by varying the concentration of the drugs (32.25–250 μM) in a phosphate buffer at pH 7.4 (Fig. S1†). As shown in Fig. S1,† the absorbance of ctDNA decreases with the addition of ART and AA without any considerable shift; however, the absorbance increased with DHAA. The observe changes clearly indicate that ART, AA, and DHAA interacted with ctDNA. However, hypochromism with an insignificant shift for the maximum absorbance at 260 nm suggest that ART and AA bind to ctDNA through groove binding. Mati *et al.* also showed a similar type of interaction of 2-(5-selenocyanato-pentyl)-6-chlorobenzo[de]isoquinoline-1,3-dione (NPOS) with calf thymus DNA (ctDNA).^36^ In case of DHAA, hyperchromic shift was ascribed to electrostatic interaction, which plays a major role in drug–ctDNA binding. Kumar *et al.* also showed electrostatic interaction between ctDNA and polyelectrolytes.^37^ As reported earlier, in the case of weaker interaction between the drug and DNA, hypochromic or hyperchromic effect was observed without any significant shift in the absorbance.^33,38^ Thus, the absorbance results indicated that ART, AA, and DHAA bind with ctDNA through weaker interactions. This result is in good agreement with the cyclic voltammetry result.

### Binding and thermodynamic parameter

The absorbance spectra of ctDNA–drug interaction were further used to determine the binding constant. The absorbance measurements of ctDNA in the absence and presence of the drugs at 260 nm were taken at two temperatures (298 and 308 K) and plotted as 1/(*A* − *A*_0_) *versus* 1/*C* through the Benesi–Hildebrand equation (double reciprocal equation).^39–41^2
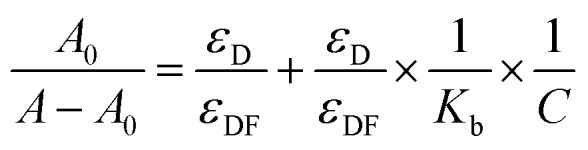
where, *A*_0_ is the absorbance of ctDNA and *A* is the absorbance of ctDNA in presence of ART, AA, and DHAA. *C* is the concentration of ART, AA, and DHAA, *ε*_D_ and *ε*_DF_ are the molar extinction coefficient for ctDNA and the drug–ctDNA complex at 260 nm, respectively, and *K*_b_ denotes the binding constant between the drug and ctDNA. As shown in Fig. S2,† we obtained a linear curve for the double reciprocal plot. The *K*_b_ was calculated through the ratio of the intercept to slope, where the correlation coefficient value (*R*^2^) was observed to be 0.99, which validates the excellence of the linear fit. *K*_b_ obtained from the Benesi–Hildebrand equation are summarized in Table 3. The lower order of binding constant, *i.e.*, 10^2^–10^3^, suggested weak binding interaction between the drugs and ctDNA. As reported earlier, the order of binding constants for the intercalators are in the range of 10^4^–10^6^ L mol^−1^ as the binding constant of EB is 4.3 × 10^5^ L mol^−1^.^42,43^ Therefore, from this result, it was confirmed that ART, AA, and DHAA bind with ctDNA by a non-intercalative binding mode, *i.e.*, groove binding and electrostatic interaction, respectively.

**Table 3:** Binding constants and thermodynamic parameters for ART, AA, and DHAA interaction with ctDNA

Drug	Temp (K)	Binding constant (*K*_b_)	Δ*H* (kJ M^−1^)	Δ*G* (kJ M^−1^)	Δ*S* (J M^−1^ K^−1^)	*R* ^2^
ART	298	1.61 × 10^3^	−59.06	−18.30	−136.79	0.9990
	303	1.08 × 10^3^		−17.61		0.9976
AA	298	1.10 × 10^3^	−20.65	−17.35	−11.07	0.9901
	303	9.60 × 10^2^		−17.30		0.9878
DHAA	298	6.59 × 10^2^	−116.81	−16.08	−338.01	0.9993
	303	3.03 × 10^2^		−14.39		0.9974

The binding constant of the drug–ctDNA interaction was further used to determine the thermodynamic parameters and the binding forces. The major non-covalent interactions that play a key role in drug–biomolecule interaction are hydrogen bond, hydrophobic interaction, van der Waals interaction, and electrostatic interaction.^44,45^ To understand the nature of binding between the drugs and ctDNA, different thermodynamic parameters such as enthalpy change (Δ*H*), entropy change (Δ*S*), and Gibbs energy change (Δ*G*) were calculated.^46^ Subsequently, to obtain the thermodynamic parameters, the UV-Visible spectroscopy data at two temperatures (298 and 308 K) were utilized by employing the following relation:^47^3
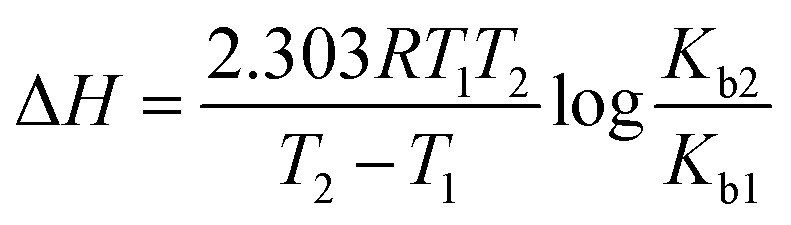

4 Δ*G* = −2.303*RT* log *K*_b_

5 Δ*G* = Δ*H* − *T*Δ*S*

where, *K*_b1_ and *K*_b2_ are the binding constants for the binding of the drugs to ctDNA at 298 and 308 K, respectively. The obtained thermodynamic values are summarised in Table 3. The negative values of Δ*G* suggest that the binding process between the drugs and ctDNA was favourable and spontaneous. Also, the negative value of both Δ*H* and Δ*S* reveals that the acting forces in drug–ctDNA binding are hydrogen bonding and van der Waals interactions.^48^ The negative value of Δ*H* suggested that the formation of drug–ctDNA complex is exothermic and enthalpy-driven. It is a well-known fact that for small molecules such as ctDNA, the interaction processes are entropically driven in intercalative binding and enthalpically driven in non-intercalative binding.^36^ Thus, the thermodynamic result showed that ART, AA, and DHAA interact with ctDNA through non-intercalative binding, *i.e.*, groove binding and electrostatic interaction.^49^

### Competitive binding displacement study

**Fig. 4:**
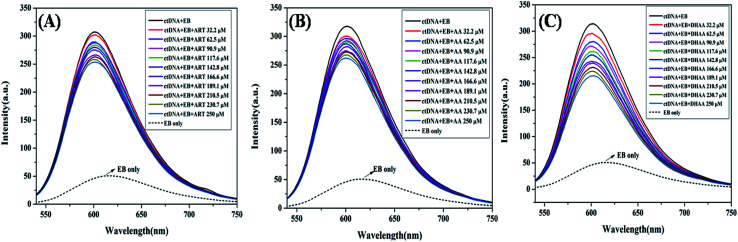
Fluorescence quenching of the EB–ctDNA complex (5 : 50 μM) at 600 nm in the absence and presence of ART (A), AA (B), and DHAA (C) at 298 K and pH 7.4.

To determine the exact mode of binding of ART, AA, and DHAA with ctDNA, we employed the competitive binding displacement assays using ethidium bromide (EB). In competitive displacement assays, the ligand is replaced by dye (EB) from DNA and it interacts with the helix of DNA in the same manner as the ligand.^50^ The changes in the fluorescence intensity of the EB–DNA complex on interaction with small molecules can be used to explore the binding mode. In the present study, we have used EB as the dye, which intercalates with the DNA base pairs. It is also a well-known fluorescence probe that is commonly used to study drug–DNA interaction. EB intercalates with the DNA base pairs on the double helix and emits intense fluorescence upon binding with DNA.^51^ The fluorescence emission of the EB–ctDNA complex was observed at 600 nm when it was excited at 467 nm. As shown in Fig. 4, on the addition of drugs in the EB–ctDNA system, the fluorescence intensity of the EB–ctDNA complex decreased in all the systems. Upon successive addition of ART and AA, the fluorescence intensity decreased up to 15%; this slight change suggested that ART/AA does not displace the EB, showing that ART and AA interact with ctDNA through groove binding. However, in the case of DHAA, there is a large decrease in the fluorescence intensity up to 35%, which shows the incomplete displacement of EB. It may be because DHAA interacts with ctDNA through electrostatic interaction and condenses the ctDNA, resulting in the formation of compact macromolecular structures that do not provide sufficient space to EB for intercalation.^37^ The competitive binding displacement result again complements the previous experimental results and confirms the non-intercalative binding mode between the drugs and ctDNA.

### Viscosity measurements

**Fig. 5:**
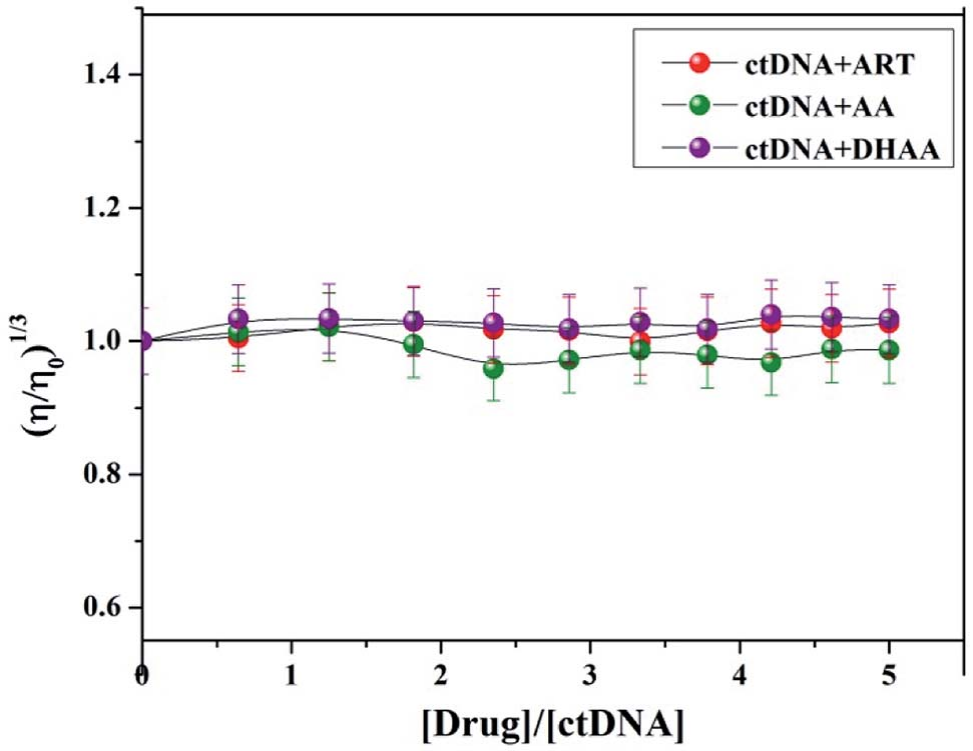
Effect of increasing concentration of ART, AA, and DHAA (32.2–250 μM) on the relative viscosity of ctDNA (50 μM).

Viscosity measurement is a very sensitive technique with the least ambiguous and most significant test for the investigation of the binding mode between small molecules and DNA.^33,36^ When a small molecule intercalates to the base pair of DNA, the length of DNA helix is enhanced, which leads to an increase in the viscosity of the system, while in the case of groove binding/electrostatic interaction, there is a minor change in the viscosity of the system due to the negligible lengthening of the DNA helix. Due to these reasons, the viscosity measurement is also a good method to confirm the binding mode; therefore, we plotted the viscosity measurement data between (*η*/*η*_0_)^1/3^*versus* [drug]/[ctDNA] and measured the viscosity change of ctDNA in the presence of different concentration of ART, AA, and DHAA (Fig. 5). It is clear from the figure that there is no significant change in the viscosity of ctDNA with increasing concentrations of ART, AA, and DHAA, which again confirmed the occurrence of groove binding between the drugs and the ctDNA system. This result was consistent with our spectroscopy result and further confirmed the non-intercalative binding mode between the drugs (ART, AA, and DHAA) and ctDNA.

### Helix melting study

**Fig. 6:**
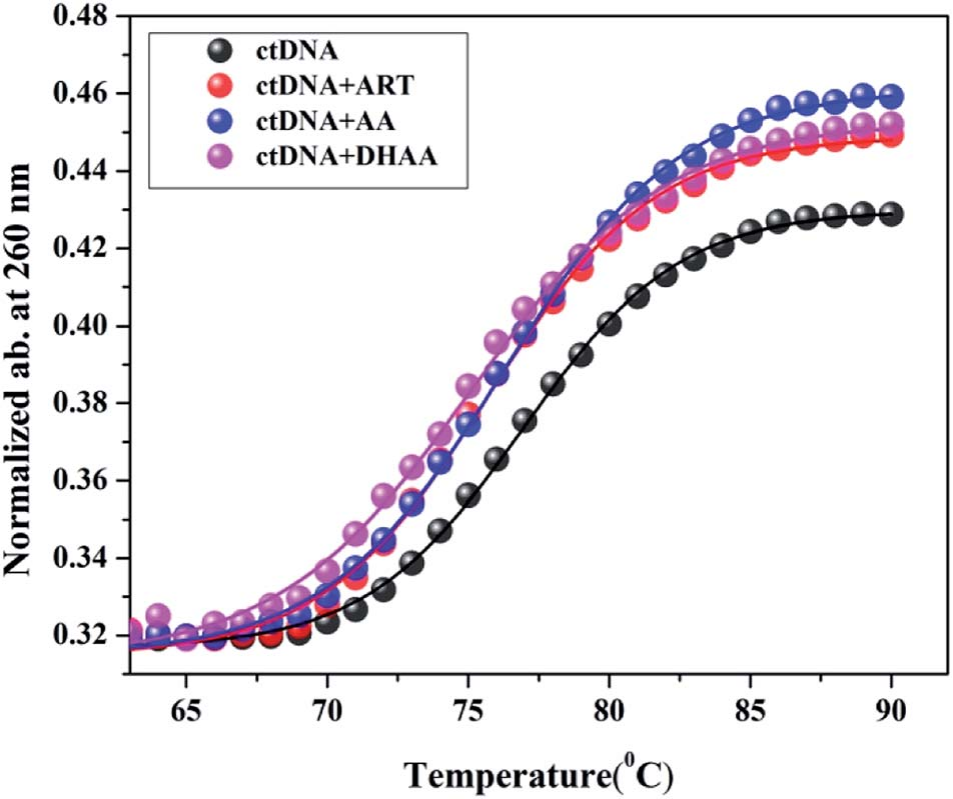
Thermal melting profiles of ctDNA (50 μM) and its complexes with ART, AA, and DHAA (250 μM).

The stability of the double-helical structure of DNA is owing to the base stacking interactions and hydrogen bonding. However, the double strand structure dissociates into single strands with increasing temperature through the melting process. The intermediate temperature at which the double helix DNA structure is 50% denatured into the single strand structure is defined as the melting temperature (*T*_m_), which is strongly related to the helical stability of DNA.^52^ The *T*_m_ was determined by as the transition midpoint of the curve for DNA absorbance at 260 nm *versus* temperature. The molar extinction coefficient of the double helical DNA structure is lower than that of the single strand structure, therefore, the absorbance increases sharply when the helix melts from the double strand to the single strand structure.^53^ The effect of *T*_m_ on the drug–DNA interactions provides valuable information about the mode of binding. For intercalative binding, a large increase in the *T*_m_ was found at about 3–8 °C due to the stabilization of the DNA double helical structure by the intercalating drug. In contrast, non-intercalative binding modes such as groove and electrostatic interaction lead to less or insignificant alteration in *T*_m_.^54,55^ In the present study, we measured the *T*_m_ value of ctDNA and the complex with ART, AA, and DHAA, which is listed in Table 4 (Fig. 6). The *T*_m_ value of the native ctDNA was calculated to be 76.91 °C, which is consistent with the literature value.^56^ As shown in Table 4, the *T*_m_ values of ctDNA with ART, AA, and DHAA do not show any substantial change, which again confirmed the non-intercalative binding mode between the drugs and ctDNA.

**Table 4:** Melting temperatures (*T*_m_) of ctDNA and its complexes with ART, AA, and DHAA

System	*T*_m_ (°C)
Native ctDNA	76.91
ctDNA + ART	75.70
ctDNA + AA	76.20
ctDNA + DHAA	75.11

### Circular dichroism study

**Fig. 7:**
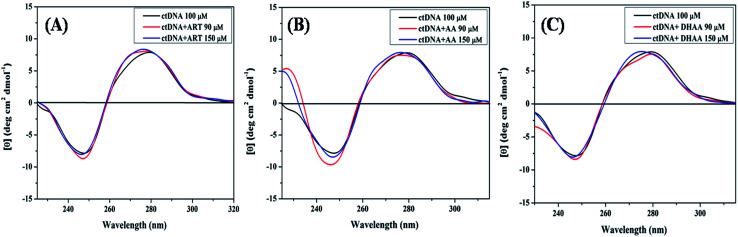
CD spectra of ctDNA (50 μM) in the presence and absence of different concentrations of ART (A), AA (B), and DHAA (C).

CD spectroscopy was also utilized to investigate the binding mechanism and the structural effect on ctDNA upon interaction with ART, AA, and DHAA. The alteration in the CD spectra of DNA leads to a change in the secondary structure of DNA on binding with drugs by non-covalent interactions. The CD spectrum of the duplex ctDNA showed two characteristic peaks of the B-DNA form, one negative band at 245 nm owing to the right-handed helicity and one positive band at 273–280 nm owing to the stacking of the base pairs. These two bands are very sensitive and informative for the drug–DNA binding mode.^57,58^ The CD spectra of both the bands show an insignificant change due to less perturbation of the base stacking and helicity bands corresponding to the groove binding and electrostatic interaction of the drug. However, the intercalative binding modes shows a significant alteration in both the positive and negative bands, stabilizing the conformation of DNA as a result.^59^Fig. 7(A–C) shows the CD spectra of ctDNA with different concentrations of ART, AA, and DHAA. It can be clearly seen that with the increasing concentration of all the drugs, both the bands in the CD spectra are unaffected, suggesting that the drugs bind with ctDNA without disturbing the base stacking and the helical structure of ctDNA, *i.e.*, groove binding.^36^ However, in DHAA–ctDNA (Fig. 7C) binding, a small shift was observed in both the negative and positive bands, which indicates the involvement of electrostatic interaction in the DHAA–ctDNA binding.^37^ In addition, a slight conformational change shows the inter-conversion of the B-form to the A-form structure of ctDNA.^54^ The CD results again confirm the non-intercalative binding mode between the drugs and DNA, which further supports our previous results.

### FT-IR spectroscopic studies

**Fig. 8:**
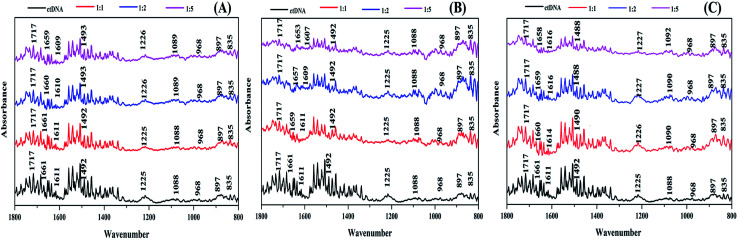
FTIR spectra of ctDNA (50 μM) in the presence and absence of different molar ratios of ART (A), AA (B), and DHAA (C).

FTIR spectroscopy is an excellent technique to determine the conformational variations that occur in DNA when it binds with ligands.^60,61^ Therefore, to understand the specific binding site and the structural effect of the ctDNA phosphate skeleton and base pairs on the binding of the drugs, we performed FTIR of free ctDNA and the drug–DNA complex with different molar ratios of ART, AA, and DHAA (Fig. 8A–C). The characteristic infrared absorption peaks of free ctDNA were observed in the region of 1800 to 800 cm^−1^ due to the ring vibrations of the nitrogenous base, phosphate stretching, and deoxyribose stretching of the DNA backbone. The nitrogenous bases of DNA, namely, guanine (G), thymine (T), adenine (A), and cytosine (C) show vibrational bands at 1717, 1661, 1611, and 1492 cm^−1^, respectively. The vibrational bands at 1225 and 1088 cm^−1^ were observed due to asymmetric and symmetric phosphate stretching. In addition, the vibrational bands at 968 cm^−1^ and 835 cm^−1^ were attributed to deoxyribose C–C and C–O stretching vibrations and the phosphodiester mode, which is the marker band of the right-handed B-form helicity, respectively.^62,63^ On the addition of different molar ratios of ART, A,A and DHAA (1 : 0, 1 : 1, 1 : 2, 1 : 5) to ctDNA, in the case of ART and AA, there is no change in the characteristic band for guanine (1717 cm^−1^) and cytosine (1492 cm^−1^) but a slight shift is observed in the adenine (1661–1659 cm^−1^ in ART, 1661–1653 cm^−1^ in AA) and thymine bands (1611–1609 cm^−1^ in ART, 1611–1607 cm^−1^ in AA), suggesting stronger interactions between the drugs and the A–T base pairs of ctDNA (Fig. 8A and B). On the other hand, no significant changes were observed in the phosphate stretching (symmetric and asymmetric) bands, indicating that ART and AA did not bind to the phosphate backbone of ctDNA.^64^ These results indicated that ART and AA bind with ctDNA through groove binding. However, in the case of DHAA, a slight change was observed in the characteristic band for adenine (1661–1658 cm^−1^), thymine band (1611–1616 cm^−1^), and cytosine (1492–1488 cm^−1^) except guanine (1717 cm^−1^), indicating a considerable change in the bands of the ctDNA base pairs (Fig. 8C). A prominent shift was also observed in the phosphate asymmetric stretching (1125–1127 cm^−1^) and symmetric stretching (1088–1092 cm^−1^) bands, which suggests that the phosphate backbone of ctDNA binds with DHAA through electrostatic interaction.^37^ The uniformity in the B-DNA marker peaks at 835 and 897 cm^−1^ with all the drugs shows the protection of the B-conformation on the binding of the drugs.^65^ The FTIR result concluded that ART, AA, and DHAA bind with ctDNA at the A–T rich region through non-intercalative binding.

### Molecular docking

**Fig. 9:**
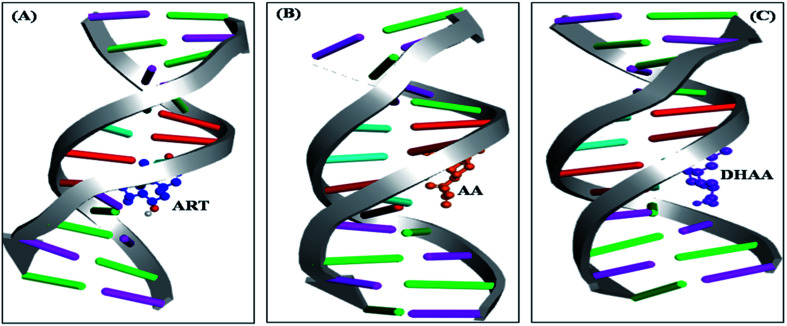
Competitive molecular docked structures of ART (A), AA (B), and DHAA (C) with ctDNA.

**Fig. 10:**
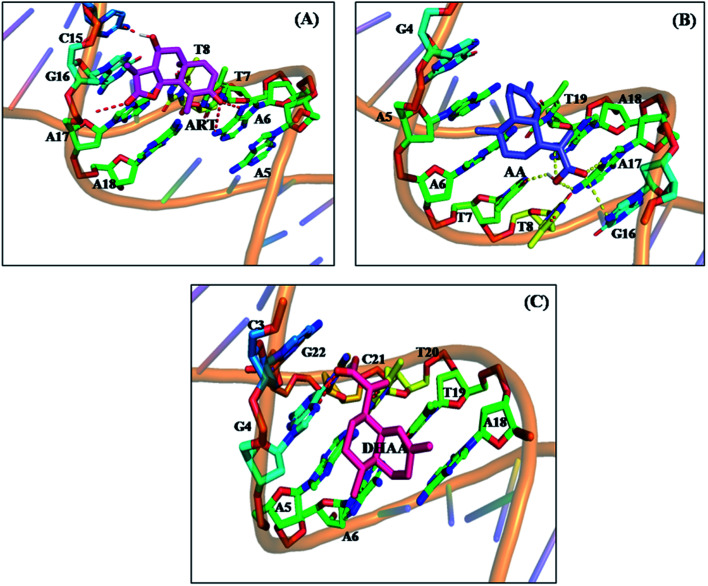
Surrounding nucleotide residues of ctDNA within 5 Å from the docked ART (A), AA (B), and DHAA (C).

The molecular docking is an imperative tool to interpret the molecular mechanism of the interaction between the DNA and drugs. It provides information about the binding location, binding site, and binging mode of the drug–DNA interaction.^66–70^ Molecular docking techniques provide an insight into the binding site and the binding mode along with the preferred orientation of the drugs inside ctDNA.^59,71^ In the present study, we performed 100 docking runs of ART, AA, and DHAA with the DNA duplex of the sequence d(CGCGAATTCGCG)_2_ dodecamer (PDB ID: 1BNA) and the most energetically favorable conformation was utilized for docking analysis. As clearly shown in Fig. 9, all the drugs (ART, AA, and DHAA) fit in contour curve of the ctDNA in the minor groove with the binding site in the A–T region, which suggests that the drugs bind with ctDNA through non-intercalative binding.^72^Fig. 10 shows that ART and AA were bound through four base pairs A–T, A–T, A–T, and C–G at the outer surface of ctDNA with hydrogen binding and van der Waals' interactions. ART was form four hydrogen bond. The two hydrogen bond forms with A6 and other two hydrogen bond with A7 and C15 residues of ctDNA, with distance 2.8, 3.3, 2.8 and 2.0 Å, respectively (refers to Fig. 10A). AA was form five hydrogen bond. The two hydrogen bond forms with A17 and other three hydrogen bond with A7, G16, and A18 residues of ctDNA, with distance 2.2, 3.5, 2.9, 2.7 and 3.6 Å, respectively (refers to Fig. 10B). However, DHAA bound through four base pairs A–T, A–T, G–C, and C–G of ctDNA with strong electrostatic interactions and two hydrogen bonds formed with the G4 (1.9 and 2.9 Å) residues of ctDNA (Fig. 10C). The relative binding energy of the docked ART, AA, and DHAA molecules with ctDNA was found to be −5.51, −3.32, and −3.75 kcal mol^−1^, respectively. The negative value of the binding energy suggested higher binding potential of ART, AA, and DHAA with ctDNA and these observed energies were analogous with the experimental results.^73^ The molecular docking result suggested the binding of ART/AA through minor groove binding and DHAA by electrostatic interaction, which shows a mutual coherence between the computational and spectroscopic techniques.

## Conclusion

The present study delivered the important depiction of the binding mechanisms of artemisinin and its biogenetic precursors with ctDNA using biophysical, electrochemical, and molecular docking methods. The cytotoxicity of ART, AA, and DHAA against three cancer cell lines, namely, H1299, A431, and HCT 116, suggest that DHAA has more cytotoxic effect than ART and AA on the colon cancer cell line. The experimental and computational results principally signify the existence of minor groove binding of ART and AA with ctDNA and electrostatic interaction in DHAA–ctDNA binding. Cyclic voltammetry and UV absorbance spectra reveal the formation of ART, AA, and DHAA complexes with ctDNA. The thermodynamic results indicated that the binding process was spontaneous and enthalpy-driven through van der Waals interaction and hydrogen bonding. Competitive displacement assays with EB confirm that ART, AA, and DHAA bind with ctDHA through non-intercalative binding, *i.e.*, groove binding and electrostatic interaction, which was further confirmed by DNA melting and CD spectroscopy. FTIR and molecular docking studies suggested that ART, AA, and DHAA bind at the A–T rich region, *i.e.*, the minor groove of ctDNA. To conclude, artemisinin and its biogenetic precursors can become promising anticancer drugs and usher a new era of applications of anticancer drug development in pharmaceutical and biomedical research.

## Experimental section

### Materials

ctDNA (96%) and artemisinin (98%) were purchased from Sigma Aldrich; artemisinic acid (98%) and dihydroartemisinic acid (99%) were obtained from Chemface and used without further purification. Ultrapure water was used to prepare all the solutions. The stock solutions of ART, AA, and DHAA were prepared in 20% ethanolic solution and diluted by 10 mM phosphate buffer (pH = 7.0). The stock solution of ctDNA was made by adding it to 10 mM phosphate buffer (pH = 7.0) and storing in 4 °C. The concentration of ctDNA was calculated by absorbance at 260 nm using the extinction coefficient *ε* = 6600 M^−1^ cm^−1^ (ref. 37) and the purity of ctDNA was determined by taking the ratio of absorbance (260/280 nm), which was found to be in the range of 1.8–1.9.

### Cell culture and MTT assay

A431 (human skin cancer cell line), HCT 116 (colon cancer cell line), and H1299 (lung cancer cell line) were obtained from National Curator of Cell Sciences (NCCS) Pune, India. The cells were cultured in DMEM (Dulbecco's Modified Eagle's Medium) added with 10% fetal bovine serum and antibiotics (2.5 μg mL^−1^ amphotericin B, 100 μg mL^−1^ streptomycin, and 100 units per mL penicillin). Cell count of 10^4^ per well were seeded in 96-well plates (150 μL per well) and maintained at 37 °C in 80% relative humidity and 5% CO_2_ in air. The cytotoxicity of artemisinin, artemisinic acid, and dihydroartemisinic acid were evaluated through MTT (3-(4,5-dimethylthiazole-2-yl)-2,5-diphenyl tetrazolium bromide) assay on A431, HCT116, and H1299. After 24 hours of incubation, the cell lines were treated with different concentrations of the drugs for 48 hours. The medium was removed after 48 hours. After that, the treated cell line was incubated in 5 mg mL^−1^ MTT (20 μL per well) solution for 4 hours. The mitochondrial enzyme formed the formazan crystals, which were solubilized in the DMSO and provided a violet color. Thus, DMSO (150 μL per well) was added into the cell line and absorbance was observed at 570 nm on a microplate reader (iMark, BIORAD, S/N 10321). The percentage of cytotoxicity was calculated through the relative absorbance of treated *vs.* untreated control cells using the following formula:^74^6
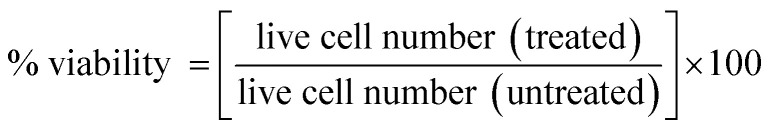


### Instrumentation and methods

The absorption spectra were measured on an Analytik Jena Specord-210 spectrophotometer in the wavelength range of 230–400 nm. The absorbance of ctDNA was observed at the fixed concentration of ctDNA (50 μM) and different concentrations of ART, AA, and DHAA (32.25–250 μM) at two temperatures of 298 and 303 K. DNA helix melting was done on the same instrument by monitoring the absorption of ctDNA (50 μM) at 260 nm with the drugs (ART, AA, and DHAA at 250 μM). The range of temperature used was from 30 °C to 90 °C. The value of the transition midpoint of the curve gave the DNA melting temperature (*T*_m_) of ctDNA and the complex.

The competitive binding studies were on a fluorescence spectrophotometer (Varian Cary Eclipse, USA). The emission spectra of ethidium bromide (EB) bound ctDNA (5 : 50 μM) were measured with different concentrations of ART, AA, and DHAA (32.25–250 μM). The wavelength range was set as 550–750 nm and the slit width for excitation and emission was kept as 5 nm. The excitation wavelength of EB-bound ctDNA was 467 nm. Cyclic voltammetry studies were carried out on a Digi-lvy (DY2100, USA) three-electrode electrochemical system. The CD and FTIR spectra were measured on a Jasco-715 spectropolarimeter and a Bruker Tensor 27 FT-IR spectrometer, respectively. The FTIR spectra of pure ctDNA (50 μM) and ctDNA were recorded in the presence of different molar ratios of ART, AA, and DHAA (1 : 0, 1 : 1, 1 : 2, 1 : 5) at 1400–1800 cm^−1^. The experimental details of cyclic voltammetry, CD, and FTIR were adopted as described in our previous work.^29,41,75,76^

The viscosity measurements were performed on a RheoSense μVISC viscometer (San Ramon, CA, USA). We observed the viscosity of ctDNA at a fixed concentration (50 μM) and with different concentrations of ART, AA, and DHAA (32.25–250 μM). The data was analyzed by plotting a graph between (*η*/*η*_0_)^1/3^ and [drug/ctDNA]. *η* and *η*_0_ denote the viscosity of ctDNA in the absence and presence of the drug.^36^

To gain an insight into ART, AA, and DHAA binding within the active site of ctDNA, we performed molecular docking using AutoDock4.2. The 3D structures of ctDNA (PDB ID: 1BNA) and the drugs (ART, AA, and DHAA) were downloaded from Protein Data Bank (PDB) and PubChem, respectively. The geometries of the drugs were optimized through Discovery Studio 2.5. To compute the possible binding site and the binding force of drug–ctDNA interaction, AutoDock4.2 was employed using Lamarckian genetic algorithm.^77^ To prepare the DNA for docking, first, the water molecule was removed and polar hydrogen atoms were added in the Autodock Tool. After that, a grid box with a grid spacing of 0.375 Å and dimension of grid size set as 40, 74, 40 along the *x*, *y*, *z* axes was generated. The docking parameter was used as follows: genetic algorithm population size: 150, number of generations: 27 000, and maximum number of energy evaluations: 2.5 × 10^6^. During the docking process, a total of 100 runs were carried out and the conformer with the lowest binding free energy was utilized for the analysis. The analyses of molecular docking were done by PyMOL (DeLano, 2004) and Discovery Studio 2.5.

## Conflicts of interest

The authors declare no competing financial interest.

## Supplementary Material

RA-010-D0RA02042G-s001
