# Supplementary material for: Comparative in vitro cytotoxicity and binding investigation of artemisinin and its biogenetic precursors with ctDNA
Source: RSC Adv. 2020 Jun 25;10(41):24203–14. doi: 10.1039/d0ra02042g (PMC9055135; doi:10.1039/d0ra02042g)
Supplement: RA-010-D0RA02042G-s001 [file RA-010-D0RA02042G-s001.pdf]

Electronic Supplementary Material (ESI) for RSC Advances.
This journal is © The Royal Society of Chemistry 2020

Supporting information

Comparative in vitro cytotoxicity and binding investigation of artemisinin and its biogenetic precursors with ctDNA

Neha Maurya[1], Khalid Imtiyaz Dar[2], M Moshahid Alam Rizvi[2], Khaled Mohamed Khedher[3], Prashant Singh[4] and Rajan Patel[1*]

[1]Biophysical Chemistry Laboratory, Centre for Interdisciplinary Research in Basic Sciences, Jamia Millia Islamia, New Delhi-110025, India.

[2]Department of Biosciences, Jamia Millia Islamia, New Delhi-110025, India.

[3]Department of Civil Engineering, College of Engineering, King Khalid University, ABHA, 6421, and Department of Civil Engineering, ISET, NABEUL, DGET, Tunisia.

[4]Department of Chemistry, ARSD College, University of Delhi, New Delhi- 110021, India.

*Corresponding author.

Tel.: +91 8860634100; fax: +91 11 26983409.

Email address: rpatel@jmi.ac.in (Dr. R. Patel)

Supplementary figures captions:

Fig. S1 UV-Vis spectra of ctDNA (50 μM) in absence and in presence of ART **(A)**, AA **(B)** and DHAA **(C)** at 298 K and pH 7.4.

Fig. S2 Double reciprocal plot between 1/A - A_0 and 1/C at tow temperatures (298 and 308 K) for ART **(A)**, AA **(B)** and DHAA **(C)**.

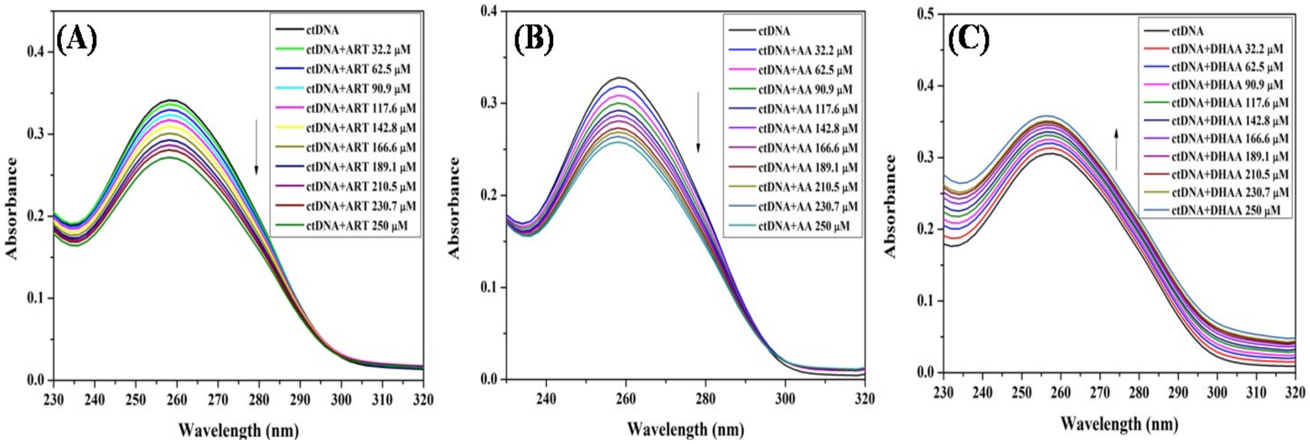

Fig. S1 UV-Vis spectra of ctDNA (50 μM) in absence and in presence of ART **(A)**, AA **(B)** and DHAA **(C)** at 298 K and pH 7.4.

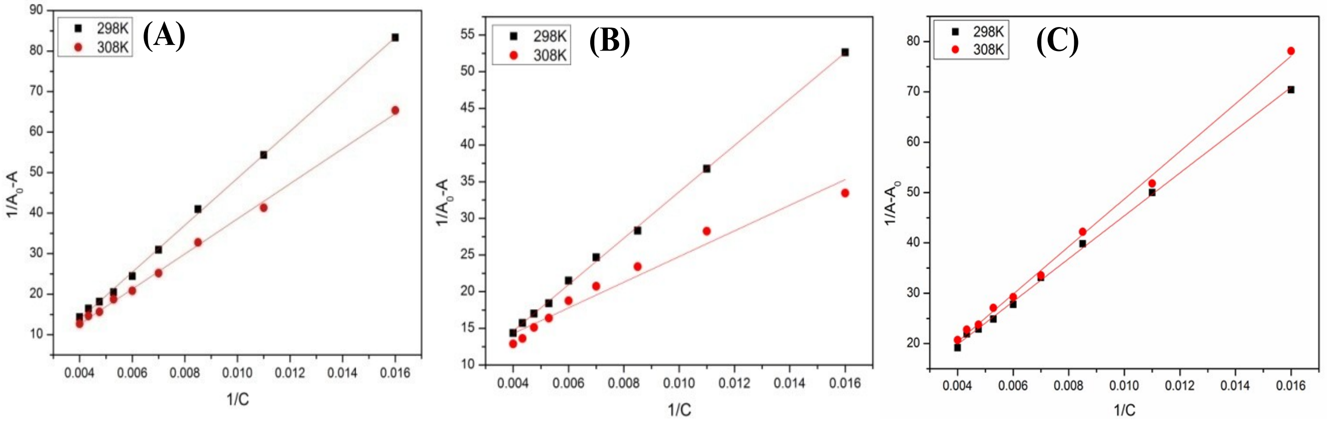

Fig. S2 Double reciprocal plot between 1/A - A_0 and 1/C at tow temperatures (298 and 308 K) for ART **(A)**, AA **(B)** and DHAA **(C)**.
